# Anatomical and Surgical Evaluation of the Common Marmoset as an Animal Model in Hearing Research

**DOI:** 10.3389/fnana.2019.00060

**Published:** 2019-06-06

**Authors:** Sho Kurihara, Masato Fujioka, Junichi Hata, Tomohiko Yoshida, Motoki Hirabayashi, Yutaka Yamamoto, Kaoru Ogawa, Hiromi Kojima, Hirotaka James Okano

**Affiliations:** ^1^Department of Otorhinolaryngology, The Jikei University School of Medicine, Tokyo, Japan; ^2^Division of Regenerative Medicine, The Jikei University School of Medicine, Tokyo, Japan; ^3^Department of Otorhinolaryngology, Head and Neck Surgery, Keio University School of Medicine, Tokyo, Japan

**Keywords:** common marmoset, diffusion tensor tractography, drug delivery, temporal bone, ear anatomy

## Abstract

Recent studies have indicated that direct administration of viral vectors or small compounds to the inner ear may aid in the treatment of Sensorineural hearing loss (SNHL). However, due to species differences between humans and rodents, translating experimental results into clinical applications remains challenging. The common marmoset (*Callithrix jacchus*), a New World monkey, is considered a pre-clinical animal model. In the present study, we describe morphometric data acquired from the temporal bone of the common marmoset in order to define the routes of topical drug administration to the inner ear. Dissection and diffusion tensor tractography (DTT) were performed on the fixed cadaverous heads of 13 common marmosets. To investigate potential routes for drug administration to the inner ear, we explored the anatomy of the round window, oval window (OW), semicircular canal, and endolymphatic sac (ES). Among these, the approach *via* the round window with posterior tympanotomy appeared feasible for delivering drugs to the inner ear without manipulating the tympanic membrane, minimizing the chances of conductive hearing loss. The courses of four critical nerves [including the facial nerve (FN)] were visualized using three-dimensional (3D) DTT, which may help to avoid nerve damage during surgery. Finally, to investigate the feasibility of actual drug administration, we measured the volume of the round window niche (RWN), which was approximately 0.9 μL. The present findings may help to establish experimental standards for evaluating new therapies in this primate model.

## Introduction

Sensorineural hearing loss (SNHL) is most commonly associated with impairments of the cochlear hair cells. Cochlear hair cell dysfunction can be caused by aging, infection, exposure to loud noises, and ototoxic drug abuse (Liberman and Kujawa, 2017). Mammalian cochlear hair cells do not spontaneously regenerate, and current treatments for chronic SNHL are ineffective. However, basic research using rodent models has contributed to the understanding of the underlying cause of hearing loss and the development of potentially effective therapies (Duan et al., 2002). Several rodent studies have indicated that direct administration of viral vectors or small compounds to the inner ear may aid in the treatment of SNHL (Li et al., 2001; Mizutari et al., 2013; Pan et al., 2017). However, recent studies have reported discrepancies in the expression of essential cochlear genes between rodents and primates (Hosoya et al., 2016a,b). In fact, some phenotypes of hereditary hearing loss cannot be recapitulated in mouse models carrying the same mutations as human patients with deafness (Plum et al., 2001; Suzuki et al., 2007). Therefore, research involving non-human primate models is required to minimize the species gap when evaluating potential interventions for hearing loss. Moreover, for the successful delivery of potential therapies, the appropriate surgical approach must be accurately defined in such a model.

The common marmoset (*Callithrix jacchus*), a New World monkey, is a valuable non-human primate model due to its small size, high reproductive efficiency, and the cross-reactivity of its cytokines and hormones with their human counterparts (Okano et al., 2012). Historically, the species has been used in the fields of toxicology, biomedicine, and neuroscience (Mansfield, 2003; Zühlke and Weinbauer, 2003; Okano et al., 2012). Numerous studies have evaluated auditory ability by assessing auditory brainstem responses (ABRs), distortion product otoacoustic emission (DPOAE), and auditory cortical physiology in marmosets (Aitkin and Park, 1993; Harada et al., 1999; Bendor and Wang, 2010; Valero and Ratnam, 2011; Johnson et al., 2016; Eliades and Tsunada, 2019; Zhu et al., 2019). Because marmosets are highly vocal and exhibit social cognition and communication patterns similar to those of humans, they are also appropriate subjects for behavioral analyses (Miller et al., 2016; Eliades and Miller, 2017). Additional studies have achieved genetic modifications in marmosets (Sasaki et al., 2009; Sato et al., 2016), rendering them a potentially useful model for hereditary hearing loss.

In the present study, we collected morphometric data related to the temporal bone in the common marmoset. Our study focused on the mastoid cavity and middle ear, which are critically related to operative manipulation. In addition, to better define important neural tracts, we utilized diffusion tensor tractography (DTT), a valuable magnetic resonance imaging (MRI) technique, to visualize neural fibers (Mori and Zhang, 2006). Generally, knowledge of the exact localization of the facial nerve (FN) is crucial for facilitating access to the inner ear without damaging the nerve. In addition to those for the FN, we also present three-dimensional (3D) images that illustrate the course of the cochlear, superior vestibular, and inferior vestibular nerves. These data provide significant information regarding the area to be avoided during surgical interventions.

Marmoset models are critical for bridging the gaps between rodents and primates in translational research. Moreover, administration of drugs or viral vectors to the inner ear may represent an effective intervention for hearing loss. While a few previous reports have described the anatomy of the common marmoset temporal bone, their assessments have favored macroscopic approaches (Borin et al., 2008; Johnson et al., 2012). The anatomical information presented herein will be particularly helpful for performing otologic surgery in the marmoset and for designing experiments to evaluate the effects of administering topical drugs or viral vectors to the inner ear.

## Materials and Methods

All experimental procedures were performed at Jikei University in Japan. Animal protocols were approved by the Institutional Animal Care and Use Committee of Jikei University (Approval no: 26-060) and performed in accordance with the guidelines of the National Institutes of Health and the Ministry of Education, Culture, Sports, Science, and Technology of Japan.

### Specimen Collection and Anatomical Measurements

The present study used the cadaverous heads of 13 [eight for dissection, three for MRI, and two for computed tomography (CT)] common marmosets (neonatal to 13 years of age) fixed with 4% paraformaldehyde (PFA)/phosphate-buffered (PBS) saline for 2 weeks.

Images were obtained using a digital camera (Nikon D5200) under a stereomicroscope (Leica M125). Structures larger than 10 mm were measured with a ruler, while smaller structures were measured using LAS X software (Leica Microsystems, Germany). We first focused on the anatomical structures and acquired the images, following which we performed the morphometric analysis.

Round window niche (RWN) volume was measured after filling the cavity with PBS.

### MRI and DTT

After fixation, specimens were stored for 2 weeks in PBS containing the contrast agent gadopentetate dimeglumine and 1 mM Magnevist (Bayer HealthCare LLC, Germany; Newman et al., 2009) to improve the signal-to-noise ratio. Specimens were scanned using a 9.4-T MRI scanner (Bruker BioSpin MRI GmbH, Ettlingen, Germany) with a maximum gradient strength of 300 mT/m on each axis. Diffusion tensor imaging (DTI) data were acquired using a 3D spin-echo sequence based on a Stejskal-Tanner diffusion preparation. The scanning parameters were as follows: repetition time (TR), 400 ms; echo time (TE), 23.8 ms; flip angle, 90°; field-of-view (FOV), 29 mm × 32 mm × 42 mm; acquisition data matrix, 138 × 152 × 200; reconstructed image resolution, 0.21 mm × 0.21 mm × 0.21 mm; *b*-value, 3,000 s/mm^2^; motion probing gradient (MPG) orientations, 30 axes; MPG duration/separation, 6/12 ms; number of averaging (NA), 1; and scan time, 72 h, 4 min, 5 s. The diffusion-tensor MRI data were analyzed using Diffusion Toolkit software (Massachusetts General Hospital, Boston, MA, USA; Wang et al., 2007). DTT was performed using TrackVis software (Massachusetts General Hospital; Wang et al., 2007). Tractography was conducted based on the Fiber Assignment by Continuous Tracking (FACT) algorithm (Mori and van Zijl, 2002) by tracking the principal eigenvector (e1) in each voxel and reconstructing it in 3D. Thus, all fiber tracts contained in each specimen were displayed. We then applied manual regions of interest (ROIs) on opposite sides of the specimen in order to selectively display specific fiber tracts (Konomi et al., 2012).

### CT

CT images of the temporal bone of an adult marmoset were obtained using the LaTheta LCT-200 *in vivo* micro-CT scanner (Hitachi-Aloka, Tokyo, Japan). The voxel size of the images was 60 or 120 μm per side (cubic voxels).

### Statistical Analyses

Statistical analysis was performed using GraphPad Prism 7.02 (GraphPad Software, Inc). Paired *t*-tests were used to evaluate differences in RWN volume. *P* values of < 0.05 were considered statistically significant.

## Results

### Surgical Dissection and Visualization of Anatomical Structures

**Figure 1:**
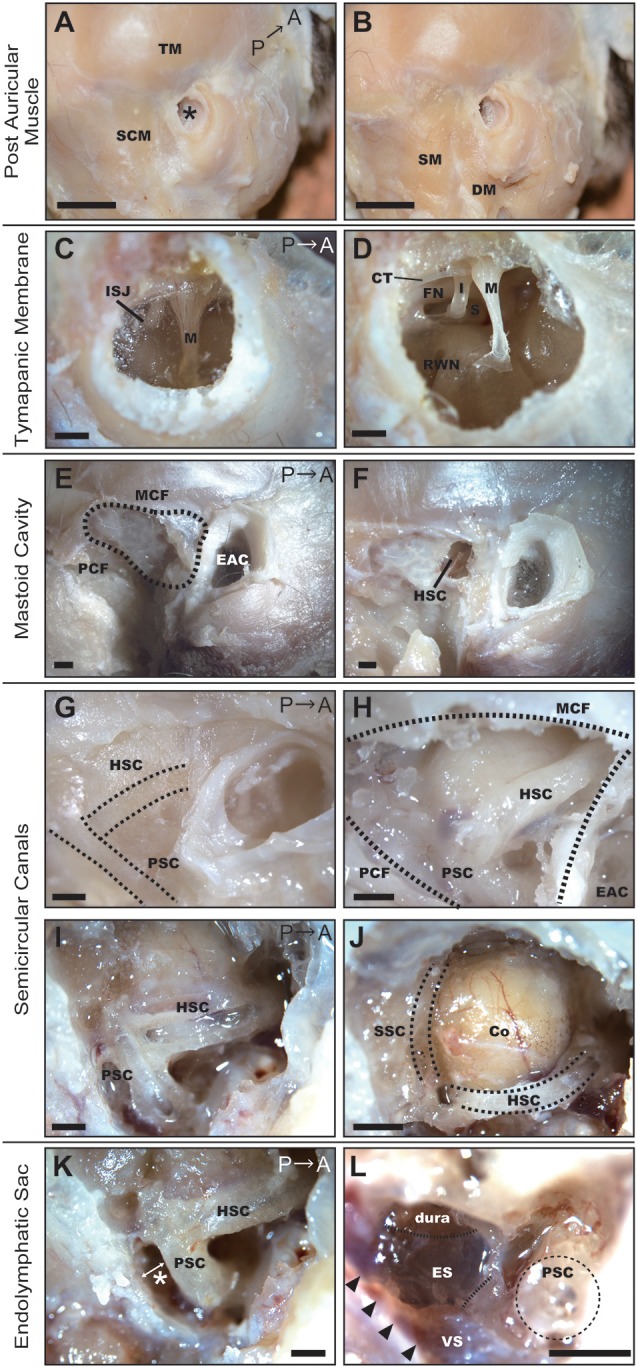
Dissection of the right temporal region of the common marmoset. **(A)** Postauricular muscles detected after the removal of skin and subcutaneous tissue. SCM, sternocleidomastoid muscle; TM, temporal muscle; Asterisk, EAC. Scale bar, 10 mm. **(B)** Muscles after the removal of the SCM. SM, splenius capitis muscle; DM, posterior belly of the digastric muscle. Scale bar, 10 mm. **(C)** Tympanic membrane as viewed from the EAC. M, malleus; ISJ, joint of incus and stapes. Scale bar, 1 mm. **(D)** The view from the EAC after extracting the tympanic membrane. M, malleus; I, incus; S, stapes; FN, facial nerve; CT, chorda tympani nerve; RWN, round window niche. Scale bar, 1 mm. **(E)** Temporal bone after removal of postauricular muscles. MCF, middle cranial fossa; PCF, posterior cranial fossa; EAC, external auditory canal. Scale bar, 1 mm. **(F)** Hole drilled on the temporal bone surface. HSC, horizontal semicircular canal. Scale bar, 1 mm. **(G)** The temporal bone of a neonatal common marmoset. Dotted lines indicate the HSC and posterior semicircular canal (PSC) as observed through the temporal bone. Scale bar, 1 mm. **(H)** Semicircular canals of a 6-month-old common marmoset. Scale bar, 1 mm. **(I)** Semicircular canals after canalostomy. Scale bar, 1 mm. **(J)** The cerebellar cortex (Co) protruding inside the HSC and superior semicircular canal (SSC). Scale bar, 1 mm. **(K)** The entry to manipulate the endolymphatic sac (ES). It was necessary to open the space between the PSC and the posterior wall of the mastoid cavity (asterisk). Scale bar, 1 mm. **(L)** ES after drilling out the PSC and stripping the petrous portion of the temporal bone. Arrowheads indicate the venous sinus (VS). Scale bar, 1 mm.

To elucidate the optimal operative approach for drug delivery to the inner ear, marmoset specimens were dissected under a microscope. In the adult common marmoset, skull height, width, and length were approximately 40 mm, 35 mm, and 45 mm, respectively. Following removal of the skin and subcutaneous tissue, the postauricular muscles were observed (Figure 1A). The temporal muscle (TM) covered most of the temporal bone surface, and the sternocleidomastoid muscle (SCM) covered almost all of the mastoid bone (Figure 1A). The insertion of the splenius capitis muscle and the posterior belly of the digastric muscle were detected below the SCM (Figure 1B).

We observed that the external auditory canal (EAC) of the common marmoset tilted forward, exhibiting an oval-shaped cross-section. The long and short diameters were approximately 3 and 2 mm, respectively. From the EAC, malleus insertion to the tympanic membrane and the joint of the incus and stapes were observed through the tympanic membrane (Figure 1C). After extracting the tympanic membrane, three ossicles (malleus, incus, and stapes) and two nerves [the FN and chorda tympanic nerve (CTN)] were detected (Figure 1D). The footplate of the stapes was situated between the prominence of the FN and the promontory. The components of the stapes (including the anterior crus, posterior crus, tendon, and head) appeared homologous to their human counterparts. We observed the RWN below the stapes.

The mastoid cavity was surrounded by the posterior wall of the EAC bone anteriorly, by the middle cranial fossa (MCF) superiorly, and by the posterior cranial fossa (PCF) posteriorly (Figure 1E). To elucidate the structure of the mastoid cavity, we obtained CT images from the specimens of an adult common marmoset (Supplementary Figure S1). The mastoid cavity was comprised of a single large cavity (antrum) and small air cells surrounding the antrum. Only a few thin bone septa were observed between the temporal bone and horizontal semicircular canal (HSC). Consistent with our CT findings, the HSC was detected after drilling a window into the temporal bone (Figure 1F). The mastoid cells of the neonatal common marmoset were not pneumatized; hence, the HSC and the posterior semicircular canal (PSC) could be visualized through the temporal bone (Figure 1G). The mastoid cavity was observed in a 6-month-old common marmoset, and the location of semicircular canals was almost analogous to that in the adult marmoset (Figure 1H). The HSC and PSC of the adult common marmoset were found to extend into the mastoid cavity (Figure 1I), and the cerebellar cortex protruded inside the HSC and superior semicircular canal (SSC; Figure 1J). The diameter of the bony canal was 0.94 ± 0.0091 mm (*n* = 4), while that of the membranous semicircular duct was 0.26–0.29 mm (*n* = 2) in the fixed condition.

The endolymphatic sac (ES) was situated between the posterior surface of the petrous portion of the temporal bone and the dura of the PCF. Visualizing the ES would have required expansion of the space between the PSC and the posterior wall of the mastoid cavity, a part of the petrous portion of the temporal bone (asterisk in Figure 1K). Although this space is large enough to manipulate in humans, it measures only 0.6–0.65 mm (*n* = 2) in the common marmoset. Thus, in the common marmoset, the PSC is relatively large when compared to the mastoid cavity. After drilling out the PSC and stripping the petrous portion of the temporal bone, the ES was observed on the dura, and its posterior portion was observed to contact the venous sinus (VS; Figure 1L).

### DTT

**Figure 2:**
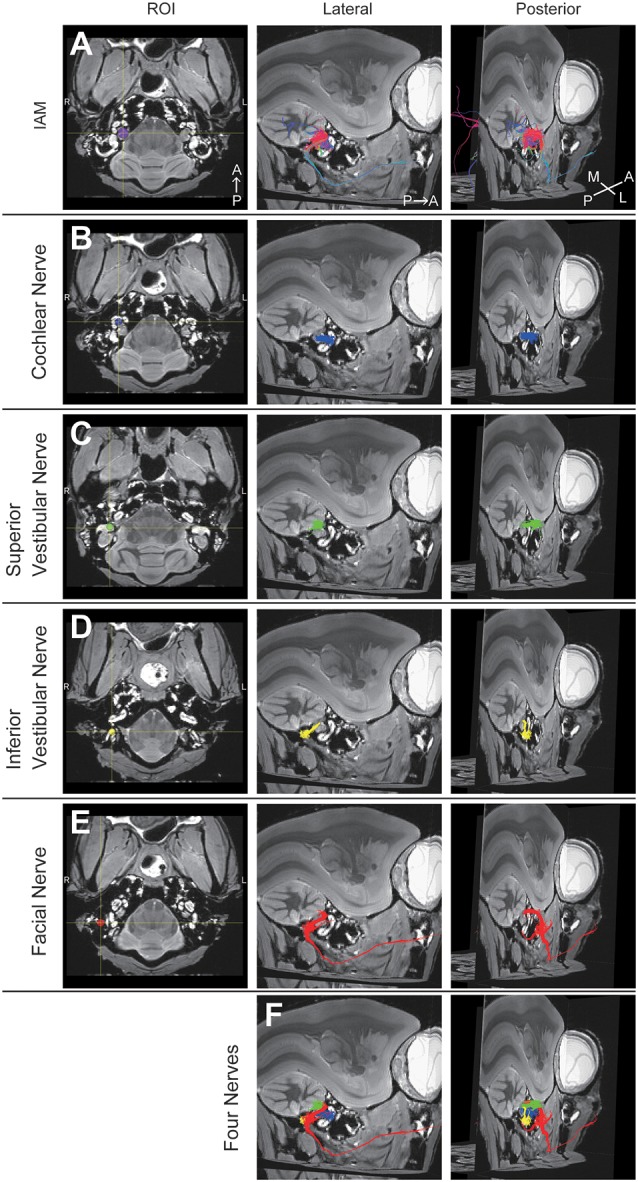
Diffusion tensor tractography (DTT) of the right temporal bone in an adult common marmoset. **(A)** Tractography with the region of interest (ROI) set on the intra-auditory meatus (IAM). The color was directionally determined. A, anterior; P, posterior; M, medial; L, lateral. **(B)** Tractography with the ROI set on the modiolus. Fibers indicating the cochlear nerve are colored in blue. **(C)** Tractography with the ROI set on the ampulla of the HSC. Fibers indicating the superior vestibular nerve are colored in green. **(D)** Tractography with the ROI set on the ampulla of PSC. Fibers indicating the inferior vestibular nerve are colored in yellow. **(E)** Tractography with the ROI set on the vertical part of the FN. Fibers indicating the FN are colored in red. **(F)** Merged image of **(B–E)**.

**Figure 3:**
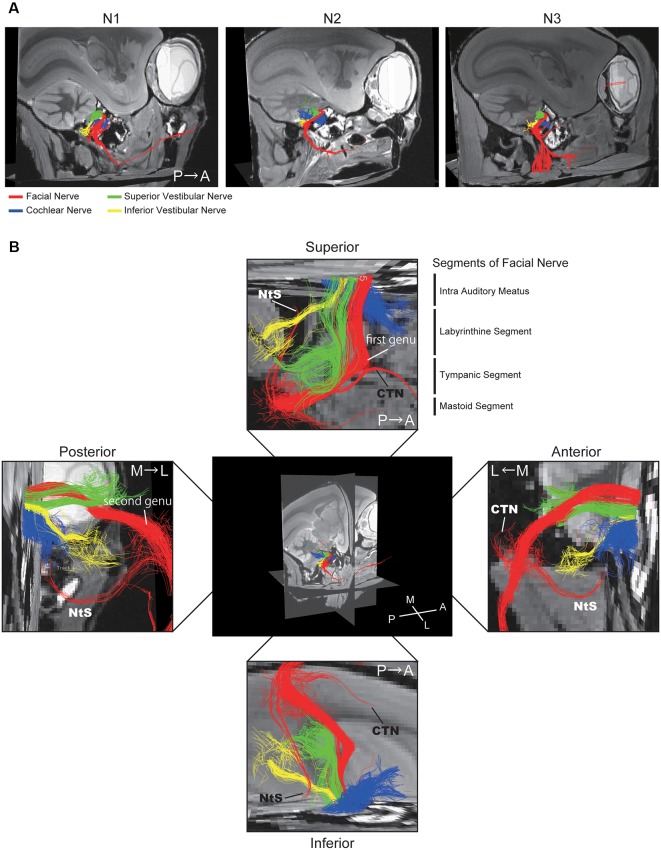
Positional relationship between the four nerves visualized using DTT. **(A)** Merged tractographic images of the four nerves obtained in three individual specimens (N1, N2, and N3). **(B)** Magnified images of the intra-auditory meatus taken from four directions; superior, inferior, anterior, and posterior. Putative segments of the FN are indicated. CTN, chorda tympani nerve; NtS, nerve to stapedius; A, anterior; P, posterior; M, medial; L, lateral.

A 3D view of the nerves within the middle ear cavity was obtained using DTT. ROIs were manually drawn based on the anatomical b0 image. Initially, fiber tracking was performed from the ROI set in the intra auditory meatus (IAM), which revealed several fiber trunks (Figure 2A). Using the same image, we identified four major nerves within the IAM: cochlear, superior vestibular, inferior vestibular, and FN. ROIs were then set on the peripheral portions of these four nerves (the modiolus, ampulla of the HSC, the ampulla of the PSC, and the vertical part of the FN) in order to visualize each nerve separately (Figures 2B–E). The tractographies of the four nerves were merged, exposing the positional relationships among the nerves in the IAM (Figure 2F). Analysis of the three specimens in a single protocol indicated a homologous course of the four nerves from the periphery to the IAM (Figure 3A). Magnified views of the IAM were obtained from four perspectives: superior, inferior, anterior, and posterior (Figure 3B). In the IAM, we observed the FN in the interior-superior region, the superior vestibular nerve in the posterior-superior region, and the inferior vestibular nerve in the posterior-inferior region. The cochlear nerve entered the IAM from the anterior-inferior region and crossed obliquely in the caudal direction. DTT of the FN revealed the positions of the first and second genua and its branches: the CTN and nerve to stapedius (NtS).

### Facial Recess

**Figure 4:**
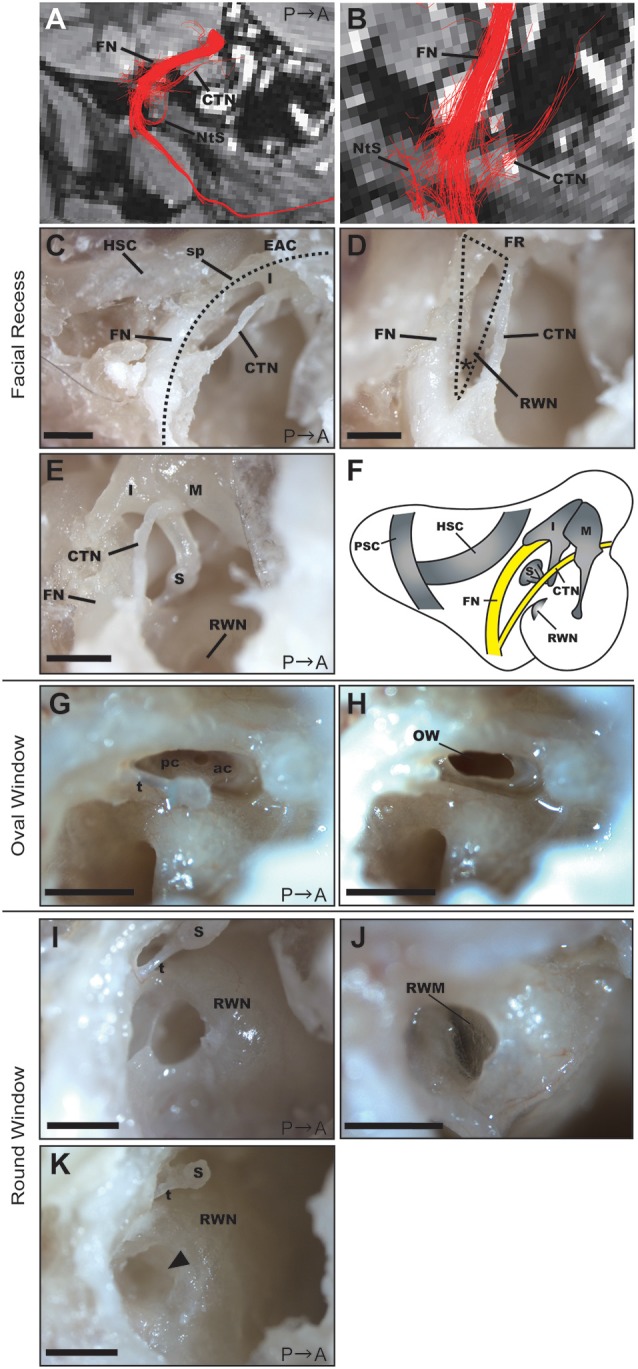
Anatomical features of the facial recess (FR), oval window (OW), and round window. **(A)** Tractography with the ROI set on the vertical part of the right FN. FN, facial nerve; CTN, chorda tympani nerve; NtS, nerve to stapedius. **(B)** Magnified view of the FR in the tractography of the FN. **(C)** Dissection of the FR after extracting the posterior wall of the EAC (dotted line). HSC, horizontal semicircular canal; I, incus; sp, short process of the incus. Scale bar, 1 mm. **(D)** Magnified view of the FR (dotted line). The asterisk indicates the angle between the FN and CTN on the bifurcation. RWN, round window niche. Scale bar, 1 mm. **(E)** Anatomical relationship between the FN and ossicles. M, malleus; I, incus; S, stapes. Scale bar, 1 mm. **(F)** Schematic representation of the right tympanic cavity and mastoid cavity. **(G)** Stapes located between the prominence of the FN and the promontory. ac, anterior crus; pc, posterior crus; t, stapedial tendon. Scale bar, 1 mm. **(H)** OW after the removal of the stapes. Scale bar, 1 mm. **(I)** RWN of a 2-year-old common marmoset. S, stapes; t, stapedial tendon. Scale bar, 1 mm. **(J)** Round window membrane (RWM) of a 2-year-old common marmoset from a different specimen. The anterior hood of the RWN was drilled out to visualize the RWM. **(K)** RWN of a 13-year-old common marmoset in which fibrous tissue plugs were observed (arrowhead). S, stapes; t, stapedial tendon. Scale bar, 1 mm.

Based on the DTT images (Figures 4A,B), we dissected the middle ear cavity to examine the relationship between the FN and bony structures. The FN crossed a bony passage known as the facial canal, which extended between the posterior wall of the EAC and HSC in the mastoid cavity, next to the stapes and RWN in the tympanic cavity (Figure 4C). The CTN ran behind the posterior edge of the tympanic membrane and then between the incus and malleus. These two nerves connected in the FN canal at the bottom of the mastoid cavity, as shown in Figure 4B. In the fixed adult common marmoset specimens, the diameter of the FN fiber was 0.38 ± 0.042 mm (*n* = 3), while that of the CTN fiber was 0.16 ± 0.003 mm (*n* = 3).

Posterior tympanotomy is the most clinically feasible procedure for approaching structures within the tympanic cavity, particularly the RWN or oval window (OW), from the mastoid cavity. We, therefore, examined the facial recess (FR), which comprises the area between the FN and CTN, to investigate whether operative manipulation can be performed *via* this recess. The width of the FR was largest just below the incus buttress, measuring 0.80 ± 0.038 mm (*n* = 3). The angle between the FN and CTN on the bifurcation was 33.3 ± 0.19 degrees (*n* = 3; asterisk in Figure 4D). The anatomical relationship of the FN and the three ossicles is shown in Figure 4E, and the schematic of the mastoid and tympanic cavities after extraction of the EAC is shown in Figure 4F.

### Oval Window and RWN

The stapedial tendon emerged from the neck of the stapes to the pyramidal eminence (Figure 4G). Unlike rodents (Buytaert et al., 2011), the common marmoset did not exhibit a stapedial artery crossing the footplate. The size of the stapes footplate was 1.04 ± 0.092 mm (*n* = 3). The OW was observed following removal of the stapes (Figure 4H).

The round window membrane (RWM) was located at the bottom of the RWN, facing posteriorly towards the FR. The RWN, a funnel-shaped depression, helps to maintain the dilution of drugs within a solvent at the site of drug administration (Figures 4I,J). We filled the RWN of dissected specimens with PBS, following which we measured the final volume of PBS within the RWN (Supplementary Table S1). RWN volume for the left ear was 0.85 ± 0.060 μL (*n* = 8), while that for the right ear was 0.914 ± 0.060 μL (*n* = 7). There was no significant difference in RWN volume between the left and right ears (*p*-value = 0.3863, *n* = 7). In the right ear of a 13-year-old specimen, a fibrous tissue plug in the RWN interfered with PBS injection; therefore, measurements could not be performed in this specimen (Figure 4K).

## Discussion

In the present study, we provided a detailed description of the anatomical features of the middle ear and mastoid cavity in the common marmoset, focusing on potential routes of drug administration to the inner ear. Two previous reports have addressed the anatomy of the temporal bone in the common marmoset. One of these studies aimed to inform the design of cochlear implants for the marmoset and reported the size of ossicles, semicircular canals, and cochlea based on CT data (Johnson et al., 2012, 2016). The other study described the macroscopic anatomical characteristics of marmoset specimens upon autopsy (Borin et al., 2008). In the current study, our anatomical description as well as the autopsy- and DTT-informed measurements have addressed several novel questions: which sites of the inner ear can be accessed during surgery, how to avoid damage to vital nerves, and what dosage of solution or a drug can be administered. These data may aid in the design of future studies involving surgical procedures for drug delivery to the inner ear.

Studies based on the data obtained from clinical surgeries and animal experiments have indicated different routes for drug administration to the inner ear, such as the round window, OW, semicircular canal, and ES. At the round window, a membrane separates the inner ear from the middle ear cavity. Thus, it is possible to approach the perilymph without opening the otic capsule from the round window. The ridge of the RWN helps to maintain the drug solution within the niche, but only a small volume of 1 μL. Therefore, it is essential to consider methods that sustain the efficacy of the drug and enhance RWM permeability while minimizing the volume of the solution administered. Mixing the drug with a carrier [e.g., poloxamer, polylactic/glycolic acid (PLGA) nanoparticles, or hydrogel] and placing it on the RWM has proven to be the most effective method for sustaining drug activity (Endo et al., 2005; Iwai et al., 2006; Nakagawa et al., 2010; Piu et al., 2011; Li et al., 2017). In addition, previous studies have reported that high osmolality solutions, benzyl alcohol, and drying of the round window can increase RWM permeability (Mikulec et al., 2008). However, in one ear of a 13-year-old common marmoset, we noticed fibrous tissue plugs in the RWN. Such obstructions of the RWN, including extraneous membranes or plugs, have also been reported in human specimens (Alzamil and Linthicum, 2000). Such obstructions are considered to be eliminated or averted by using a collagenase solution (Xia et al., 2012) or by surgical manipulation. Although direct injection of the drug into the perilymph with penetration of the RWM is one alternative method of administration, it remains unknown whether fenestration of the RWM can induce hearing loss or vestibular dysfunction in humans.

Posterior tympanotomy can be useful for delivering drugs to the round or OWs without manipulating the tympanic membrane, as this may otherwise lead to conductive hearing loss. Similar to humans, common marmosets have a mastoid cavity that communicates with the tympanic cavity only through the aperture of the mastoid antrum. This suggests that the postauricular approach (opening up the bulla and manipulating the structure of the middle ear cavity), which is commonly used in rodent models (Akil et al., 2012, 2015), cannot be utilized in the common marmoset. Although the scale differs, posterior tympanotomy should be thus be performed in the common marmoset. The FR is only 0.8 mm wide in the common marmoset, requiring a 0.6-mm diameter diamond bar to open the window without damaging the FN or CTN. Prior to surgery, DTT can be used to inform the construction of the operative image and help to avoid damaging these nerves.

Recent studies have utilized a trans-oval-window approach for drug administration in murine models (King et al., 2013; Sircoglou et al., 2015). Interestingly, King et al. (2013) reported that gentamicin spread more rapidly in the inner ear when applied to the stapes footplate than when applied to the RWM. Although the trans-oval-window approach is feasible, care must be taken not to touch the stapes, as this may induce conductive hearing loss or SNHL. In the common marmoset, the stapes was situated deeply between the FN canal and the promontory, making it difficult to access.

While the basic structure of the semicircular canals in the common marmoset was similar to that in humans, it differed in that the canals occupy a larger part of the mastoid cavity. Because the HSC and PSC can be easily located in the mastoid cavity, drug administration can be achieved directly after canalostomy. The membranous semicircular duct is less than 0.3 mm in diameter when in the fixed condition and requires a 31-gauge (or thinner) needle for puncture. Care must be taken during manipulation in order to avoid damage to the region of the cerebellar cortex that protrudes inside the HSC and SSC. A previous study reported that direct injection of adenovirus *via* the semicircular canal in mice led to transduction, predominantly in the vestibular organ (Kawamoto et al., 2001). Other mouse studies demonstrated that viral vectors injected from the semicircular canal diffuse into the cochlea, introducing transgene expression in cochlear hair cells (Pfannenstiel et al., 2009; Guo et al., 2017). Therefore, further studies are required to evaluate the efficiency of injection from the semicircular canal and diffusion of the injected solution into the inner ear canal in the common marmoset.

The ES connects to both the vestibular organ and cochlea through the endolymphatic flow and is thought to play an absorptive role in the endolymphatic organs. Inner ear transgene expression has been reported after adenoviral vector injection in the ES in a mouse model (Yamasoba et al., 1999). The transgene was detected not only in the ES and vestibular end organs but also in the cochlea and stapes. Therefore, this approach seems to be viable for diffusing drugs into the inner ear, especially for targeting the sensory epithelium located in the endolymph. In patients with uncontrollable vertigo, the ES can be surgically manipulated during ES decompression. However, in the common marmoset, the procedure differs since the pathway to the ES is very narrow. The space between the PSC and posterior wall of the mastoid cavity is smaller than 0.7 mm. Drilling the space can potentially damage the VS that runs directly beneath the posterior wall of the mastoid cavity.

Hence, in the present study, we analyzed the anatomical features of the tympanic and mastoid cavities of the common marmoset; summarized the size of anatomical features related to inner-ear drug delivery in humans, common marmosets, and mice (Supplementary Table S2); and listed the advantages and disadvantages of each potential route of drug administration to the inner ear (Supplementary Table S3).

When using the common marmoset model for translational research, we must consider not only the feasibility of the approach to the inner ear without causing any complications but also the applicability of the administration route to clinical settings. Our results indicated that posterior tympanotomy and drug delivery *via* the RWN represented the most suitable protocol. We are planning a future *in vivo* study to verify the expansion of the drug or viral vector into the inner ear of the common marmoset, which will likely have important implications for translational medicine (e.g., cochlear hair cell regeneration, which has been accomplished in rodents and guinea pigs; Bermingham et al., 1999; Chen et al., 2002; Izumikawa et al., 2005; Mizutari et al., 2013; Mittal et al., 2017).

## Conclusion

In this study, we explored the anatomical characteristics of the middle ear and mastoid cavity in the common marmoset, focusing on how such features relate to drug delivery to the inner ear. DTT-based 3D imaging improved our understanding of vital nerve routes in this model. The information presented herein will help to establish standards for future evaluations of novel therapies using this primate model.

## Data Availability

All datasets generated for this study are included in the manuscript and/or the Supplementary Files.

## Ethics Statement

This study was carried out in accordance with the recommendations of The Institutional Animal Care and Use Committee of the Jikei University (Approval no: 26-060).

## Author Contributions

SK, MF, KO, HK, and HO conceived the research idea. SK, TY, MH, and YY designed and performed the experiments. JH analyzed the MRI data. SK, MF, and HO wrote the manuscript.

## Conflict of Interest Statement

The authors declare that the research was conducted in the absence of any commercial or financial relationships that could be construed as a potential conflict of interest.

## Supplementary Material

FIGURE S1 Computed tomography images of the temporal bone. **(A)** Axial and coronal view of the temporal bones of two adult specimens. A, anterior; P, posterior; R, right; L, left, Cr, cranial; Ca, caudal. **(B)** Sequential magnified images of the middle ear cavity. M, malleus; I, incus; HSC, horizontal semicircular canal; PSC, posterior semicircular canal.

TABLE S1 Round window niche volume in eight specimens. Note that the round window niche in the right ear of a 13-year-old specimen was filled with fibrous tissue plugs.

TABLE S2 Comparisons of the anatomical features of the inner ear in humans, common marmosets, and mice.

TABLE S3 Advantages and disadvantages of each potential route of drug administration to the inner ear in common marmosets.
