# Supplementary figures and images for: Anatomical and Surgical Evaluation of the Common Marmoset as an Animal Model in Hearing Research
Source: Front Neuroanat. 2019 Jun 6;13:60. doi: 10.3389/fnana.2019.00060 (PMC6563828; doi:10.3389/fnana.2019.00060)

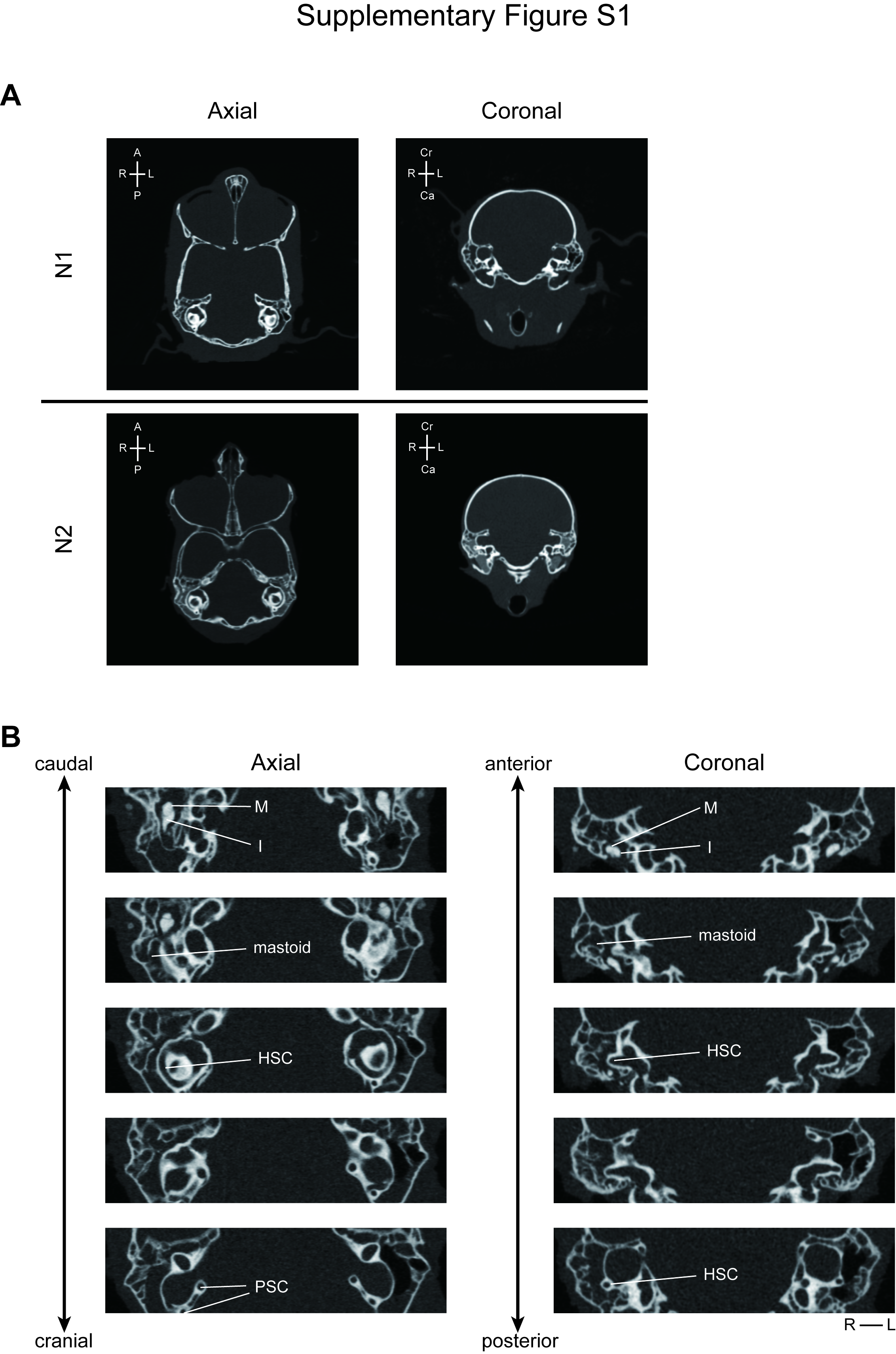

Supplement: FIGURE S1 — Computed tomography images of the temporal bone. (A) Axial and coronal view of the temporal bones of two adult specimens. A, anterior; P, posterior; R, right; L, left, Cr, cranial; Ca, caudal. (B) Sequential magnified images of the middle ear cavity. M, malleus; I, incus; HSC, horizontal semicircular canal; PSC, posterior semicircular canal. [file Image_1.TIF]
