# Supplementary material for: Anatomical and Surgical Evaluation of the Common Marmoset as an Animal Model in Hearing Research
Source: Front Neuroanat. 2019 Jun 6;13:60. doi: 10.3389/fnana.2019.00060 (PMC6563828; doi:10.3389/fnana.2019.00060)
Supplement: TABLE S1 — Round window niche volume in eight specimens. Note that the round window niche in the right ear of a 13-year-old specimen was filled with fibrous tissue plugs. [file Data_Sheet_1.PDF]

Supplementary Table S1

Age	Left (μl)	Right (μl)
newborn	0.6	0.8
1 year	0.8	0.9
2 years	0.8	0.8
2 years	0.9	1.2
2 years	1.2	1
2 years	0.9	0.9
2 years	0.8	0.8
13 years	0.8	NA
Mean,SEM	0.85, 0.060	0.91, 0.055
p value	0.742	
