# Supplementary material for: Anatomical and Surgical Evaluation of the Common Marmoset as an Animal Model in Hearing Research
Source: Front Neuroanat. 2019 Jun 6;13:60. doi: 10.3389/fnana.2019.00060 (PMC6563828; doi:10.3389/fnana.2019.00060)
Supplement: TABLE S2 — Comparisons of the anatomical features of the inner ear in humans, common marmosets, and mice. [file Data_Sheet_2.pdf]

Supplementary Table S2

	Common Marmoset	Human	Mouse
Number of cochlear turns	2.84[a]	2.76-2.84[b]	1.5[h]
Volume of round window niche (mm^3)	0.88	4.65[c]	NA
Obstruction of round window niche	Fibrous plug: 12.5% (n = 1 / 8)	Fat plugs: 1.5% [d]<br>Fibrous plugs: 10.4%<br>Extraneous RWM: 21.3%	not confirmed[h]
Foot-plate size [length×width (mm) / area (mm^2)]	1.14×0.55[a] / NA	2.64-3.36×1.08-1.66[e] / 2.65-3.75[f]	NA / 0.093[i]
Semilunar-canal size [width (mm)]	0.94	1.2-1.4[g]	0.2[h]
Ear-drum size [length×width (mm) / area (mm2)]	4.61×4.71[a] / NA	8.5-10×8-9 / 85[e]	NA / 2.75[i]

[a] Johnson et al., 2012
[b] Borin et al., 2008
[c] Takahashi et al., 1989
[d] Alzamil and Linthicum, 2000
[e] Adams and Liberman, 2010
[f] Wever and Lawrence, 1954
[g] Beck and Bader, 1963
[h] Jero et al., 2001
[i] Saunders and Summers, 1982
