# Supplementary material for: Anatomical and Surgical Evaluation of the Common Marmoset as an Animal Model in Hearing Research
Source: Front Neuroanat. 2019 Jun 6;13:60. doi: 10.3389/fnana.2019.00060 (PMC6563828; doi:10.3389/fnana.2019.00060)
Supplement: TABLE S3 — Advantages and disadvantages of each potential route of drug administration to the inner ear in common marmosets. [file Data_Sheet_3.pdf]

Supplementary Table S3

| Route | Advantage | Disadvantage |
| --- | --- | --- |
| **Semicircular canal**<br>*Kawamoto et al., 2001*<br>*Pfannenstiel et al., 2009*<br>*Guo et al., 2017* | ・Easy<br>・Used clinically | ・The expansion to the cochlea is controversial |
| **Endolymphatic sac**<br>*Yamasoba et al., 1999* | ・Direct injection to endolymph<br>・Used clinically | ・Difficult |
| **Oval window**<br>*King et al., 2013*<br>*Sircoglou., 2015* | ・Possibility of greater expansion than the trans-round-window membrane | ・Difficult<br>・Risk of hearing loss |
| **Round window (direct injection)**<br>*Akil et al., 2012*<br>*Askew et al., 2015* | ・Secured expansion to the cochlea | ・Risk of hearing loss |
| **Round window (trans membrane)**<br>*Nakagawa et al., 2010*<br>*Li et al., 2001*<br>*Mizutari et al., 2013* | ・Used in a clinical trial<br>・Clinically acceptable | ・Volume is restricted to 0.9 μL |
